# One-stage release of congenital constriction band in lower limb from new born to 3 years

**DOI:** 10.4103/0019-5413.61978

**Published:** 2010

**Authors:** Sakti Prasad Das, PK Sahoo, RN Mohanty, SK Das

**Affiliations:** Swami Vivekananda National Institute of Rehabilitation Training and Research, Olatpur, Bairoi, Cuttack, Orissa-754 010, India

**Keywords:** Amniotic band, congenital constriction band, one-stage release

## Abstract

**Background:**

Congenital constriction band is the most common cause of terminal congenital malformation of a limb and lymphoedema. Superficial bands do not need any treatment, but deeper bands are managed with excision and Z-plasty. The circumferential bands are released in two to three stages to prevent vascular compromise. The purpose of this study was to present the outcome of one-stage release.

**Materials and Methods:**

Nineteen children, 12 boys and 7 girls, with 24 congenital constriction bands constituted the clinical material. The mean age at presentation was 57 days (range 12 hours to 3 years) Band was unilateral in 14 and bilateral in five limbs. In unilateral cases, right side was involved in nine cases and left side in five. The constriction band is seen at the junction of middle and distal third. The patients having constriction bands in lower limbs and age less than 3 years were included in the study. One stage circumferential release of congenital constriction band was performed. Our youngest patient was operated at the age of six months. Club feet, (n=8) and lymphedema (n=7) were associated anomalies. Club feet and band were released in one stage in three limbs. The results were evaluated by criteria described by Joseph Upton and Cissy Tan.

**Results:**

There were 18 excellent, six satisfactory results. No wound problem occurred. No vascular compromise was noted during or after the procedure. On follow-up, distal swelling reduced.

**Conclusions:**

One-stage circumferential release of congenital constriction band in lower limbs with or without lymphodema is a safe and easy procedure.

## INTRODUCTION

Congenital constriction band syndrome, also called amniotic band syndrome, affects approximately 1 in 1200 to 1 in 15000 live births.^1^,^2^

Up to 50% cases have other anomalies including cleft lip, cleft palate and club foot deformity,^1^–^5^ syndactyly, peripheral nerve defects, distal lymphoedema 6–10 and intra uterine lymphodema.^1^ New born babies may present with superficial or deep constriction bands-either unilateral or bilateral involving limb.^1^

Superficial bands do not need any type of treatment as it rarely causes any vascular problems. However, deeper bands need management with excision and multiple Z -plasty procedures to relieve distal swelling and lymphoedema.^1^ It is very difficult to treat circumferential bands because there are either wound complications or cases of recurrence of contracture. Surgical planning and procedures are also specialized. They are released in two to three stages to prevent vascular complications of distal parts. This concept, first propagated by Stevensons, is advocated by many surgeons.^2^,^4^–^6^,^8^,^9^,^11^–^13^ Several reports of one-stage release of congenital constriction band have been reported in literature 1,15–18 with good results. We report a series of one-stage release of a circumferential constriction band in few cases.

## MATERIALS AND METHODS

24 congenital constriction bands in 19 children were included in this study. There were 12 boys and seven girls, We included patients having deep constriction bands in lower limb and age less than 3 years. Associated upper limb constriction bands were present in 5 cases which were superficial and did not require any treatment. Bands were unilateral in 14 and bilateral in five limbs. In unilateral cases the right side was involved in nine cases and left side in five. Lymphoedema was present in seven limbs. The mean age of patients at presentation was 57 days (range 12 hours to 3 years). Twelve patients reported within one year of birth and one child on the first day of birth. Club feet were associated with eight limbs in 19 limbs constriction band was at junction of middle and lower third and in five cases it was in upper third. All the cases were followed from two to seven years (mean 5 years) and were operated by the first author.

The depth of the ring was classified as superficial or deep. Superficial rings have only a circumferential mark over the limb without any distal swelling. All our cases had deep rings. In all our cases distal swelling was present. X-rays were done in all the cases to exclude pseudo arthrosis of tibia. None of the patients had pseudoarthrosis.^14^ We have not done angiography to study the vascular status of the limb.

### Operative procedure

**Figure 1:**
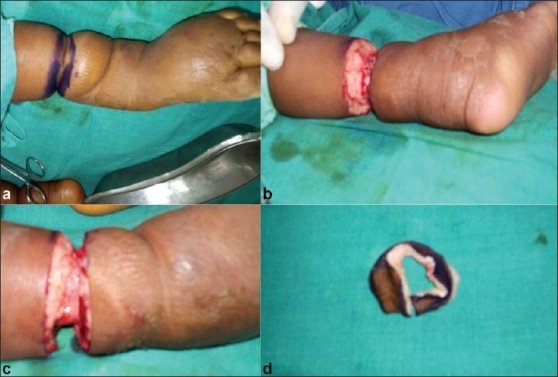
Clinical photographs showing (a) Pre-operative photograph marked with marker pen. (b and c) Per operative photograph after release of the constriction band (d) Band after excision

After preparation of the part, marking ink is applied to the band circumferentially to assess the apposition after the release. This marking ink gives the surgeon an idea about the amount of skin to be excised from both sides of the band. The incision on both sides of the band normally takes two mm of normal tissue. Fibrous bands along with normal tissue is released in a circumferential manner [Figure 1a–d]; both the proximal and distal parts of limb from the constriction band, are approximated to each other. Then mattress suture is applied. This is followed by soft dressing. Stitches are removed after 10 days. The dressing is changed thrice in 10 days. Circulation to the leg and foot are assessed by capillary refill and color of the skin. Postoperatively, the part is massaged distal to proximal (centripetal) to reduce edema. Ankle foot polypropelene orthosis is applied in club feet cases to maintain correction and prevent recurrence.

In all our patients, the surgical wound healed uneventfully and the children were discharged from hospital in 10 days.

Of the eight cases associated with club feet, three were operated in the same sitting. After release of constriction band, posterior medial soft tissue release was (PMR) done with the classical single incision technique in the same sitting. There were no wound complication of club feet cases operated in the same sitting. The remaining five cases were operated upon after four to six weeks.

**Figure 2:**
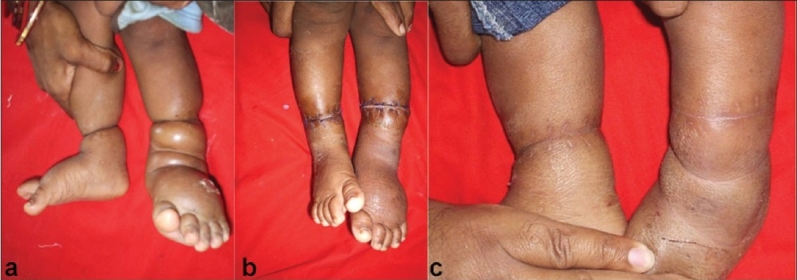
Clinical photographs showing (a) Congenital constriction band in both lower limbs, (b) Three weeks post-operative follow up, (c) One year post operative follow-up of same patient

The patients, X-rays and photographs were evaluated at 6 monthly intervals between one to 10 years after constriction ring release [Figure 2a–c]. Anteroposterior (AP) and lateral view X-rays were done to observe various angles of correction in club feet cases and assess limb length (shortening of tibia and narrowing of tibia at band site and future limb length problems) problems of tibial component. Because in some cases tibia is found to be short and narrowing at band site. All evaluations were based on objective clinical examinations. The results were evaluated as per criteria of Joseph Upton and Cissy Tan^4^ [Table 1].

**Table 1:** Joseph Upton and Cissy Tan criteria for evaluation of results

Excellent	Normal contour, inconspicuous scars, no depression and no distal swellings
Satisfactory	Improved contour, noticeable scars with slight depression over site of original constriction and no distal swelling
Poor	No contour improvement, conspicuous incision with depressions or recurrence of original deformity

## RESULTS

Twenty cases were excellent and four cases were satisfactory based on Cissy Tan scoring.^4^

There was no skin necrosis or wound healing problem. There was no circulatory disturbance in either with band or with band associated with club feet in the immediate post operative period. Neurovascular problems are the most important complications any surgeon is afraid of but with little care these things can be avoided. The swelling of the leg was markedly reduced in subsequent follow-ups. No wound complications were noted in club feet cases operated in same sitting with band release.

There were no problems related to the skin, range of movement of ankle, and leg length discrepancy at final follow up. There was reduction of distal swelling on subsequent follow up. Club feet cases were managed post operatively by ankle foot orthosis.

## DISCUSSION

Most studies favor the theory enunciated by Torpin^19^ that rupture of amnion permits the fetus from entering the chorionic cavity with tethering of extremities and resulting oligohydramnios produce club feet and other positional anomalies. Ruptured amnion act as a band around the limb leading to constriction band in the limb or amputation in uterus.

The part distal to the circumferential ring may be swollen and distal circulation may be compromised. Disruption of circulation results in amputation in uteri. Lymphodema also has been reported in most of the cases. Neurological deficits distal to the constriction band have been reported.^5^,^7^,^9^,^14^,^16^,^18^ The etiology may be axonotemesis or neurotemesis caused by direct pressure from the constriction band. It is beyond of scope from this chapter to study the neurological deficits.

The concept advocated by Stevenson^20^ was to release the constriction ring in multiple stages with the aim to prevent circulatory disturbance. Further studies on circulation to the skin flaps^21^ found that blood supply to the skin is primarily from the musculo cutaneous arteries that directly penetrate the subcutaneous and cutaneous tissue from underlying muscles. This is very important support to single stage contracture release. This observation confirms that there will be no wound healing problems and venous obstruction. Removal of band actually facilitates blood circulation to muscles in a severely involved limb. Deep vessels are not damaged by our surgical procedures.

The aim of the surgery for a congenital constriction band is complete excision of the constricted tissue to minimize the risk of recurrence and preserve circulation. Hence, one to two mm of normal skin and subcutaneous tissue was excised along with dense fibrous tissue to avoid future indentation. All dense fibrous tissue needs to be excised completely.^11^,^14^ Apposition of normal tissue results in good healing with maintenance of contours.

One-stage release does not place the underlying neurovascular structures at greater risk than multiple staged Z plasties. There was no skin necrosis or wound healing problem. Before accepting this method we had tried a few cases with multiple staged surgeries. It was found that the remaining half of band has a tethering effect, which tries to pull the released side causing increased chance of recurrence. An additional operation, anesthesia and ultimately financial burden could hence be avoided by using one stage release.

In conclusion, one-stage release of congenital constriction band results in one-stage correction, avoids repeat surgery, requires easy postoperative care, reduces financial burden and saves time of patient. The improvement noted at six months was maintained at six years.
